# Spatial Frequency Selectivity Is Impaired in Dopamine D2 Receptor Knockout Mice

**DOI:** 10.3389/fnint.2017.00041

**Published:** 2018-01-15

**Authors:** Bruno Oliveira Ferreira Souza, Mira Abou Rjeili, Clémentine Quintana, Jean M. Beaulieu, Christian Casanova

**Affiliations:** ^1^Laboratory of Visual Neuroscience, Optometry School, University of Montreal, Montreal, QC, Canada; ^2^Department of Pharmacology and Toxicology, University of Toronto, Toronto, ON, Canada

**Keywords:** dopamine receptor, cortical maps, optical imaging, primary visual cortex, mouse model

## Abstract

Dopamine is a neurotransmitter implicated in several brain functions, including vision. In the present study, we investigated the impacts of the lack of D2 dopamine receptors on the structure and function of the primary visual cortex (V1) of D2-KO mice using optical imaging of intrinsic signals. Retinotopic maps were generated in order to measure anatomo-functional parameters such as V1 shape, cortical magnification factor, scatter, and ocular dominance. Contrast sensitivity and spatial frequency selectivity (SF) functions were computed from responses to drifting gratings. When compared to control mice, none of the parameters of the retinotopic maps were affected by D2 receptor loss of function. While the contrast sensitivity function of D2-KO mice did not differ from their wild-type counterparts, SF selectivity function was significantly affected as the optimal SF and the high cut-off frequency (*p* < 0.01) were higher in D2-KO than in WT mice. These findings show that the lack of function of D2 dopamine receptors had no influence on cortical structure whereas it had a significant impact on the spatial frequency selectivity and high cut-off. Taken together, our results suggest that D2 receptors play a specific role on the processing of spatial features in early visual cortex while they do not seem to participate in its development.

## Introduction

Dopamine (DA) is a neurotransmitter that plays a central role in several brain functions such as motor control, cognition and motivated behaviors. Dopamine modulates neuronal activity through a set of G-protein coupled receptors divided in two functionally distinct groups based on their effects on the intracellular levels of cyclic AMP, D1-class (D1 and D5) and D2-class (D2S, D2L, D3, and D4) receptors (Witkovsky, 2004; Beaulieu and Gainetdinov, 2011).

Dopamine receptors play an important role in the development and function of several brain regions (Money and Stanwood, 2013). Disruption of the DAergic system gives rise to several debilitating conditions such as Parkinson's disease and Schizophrenia (Howes and Kapur, 2009; Gama et al., 2014). Alterations of visual perception are symptoms frequently reported in those diseases (Bodis-Wollner, 2009; Green et al., 2009; Botha and Carr, 2012). For example, Parkinson's patients experience a variety of visual deficits such as reduced visual acuity, contrast sensitivity, color perception and are prone to visual hallucinations (Büttner et al., 1996; Bodis-Wollner, 2009; Gama et al., 2014). Such spectrum of symptoms indicates that the DAergic system participates in multiple levels of neural processing of visual information. As such, numerous studies have investigated the presence and functional implications of the DAergic system in several key areas of the visual system.

In the retina, the release of dopamine from a subset of amacrine cells is involved in light adaptation, contrast sensitivity and spatial frequency (SF) selectivity (Bodis-Wollner and Tzelepi, 1998; Witkovsky, 2004; Huppé-Gourgues et al., 2005) as well as in non-visual processes such as the control of circadian rhythm and ocular growth (McCarthy et al., 2007; Feldkaemper and Schaeffel, 2013). Studies in primates revealed that the inactivation of D2-class receptors alters the SF tuning of ganglion cells (Tagliati et al., 1994) and the blockage of these receptors in humans reduced the signal amplitude of pattern ERG in a dose-dependent manner (Stanzione et al., 1995). A recent study investigated the impact of the absence of D1 and D2 dopamine receptors in respective knockout mice models (Lavoie et al., 2013). Interestingly, the lack of functional D2 receptors has little effect on the electroretinogram (ERG) of knockout mice suggesting that this receptor plays a minor role on DA modulation of retinal physiology in mice.

D2-class receptors are also found in the dorsal lateral geniculate nucleus (dLGN) (Khan et al., 1998) and are directly implicated in the modulation of excitatory glutamatergic synapses of relay neurons (Govindaiah and Cox, 2006). Interestingly, the local injection of D2 agonists influenced the contrast response gain of relay neurons from dLGN (Zhao et al., 2001). It is thus likely that these changes are reflected at the level of the recipient cells in the primary visual cortex.

While D2 receptors are also present in layers IV and V of the visual cortex (in primates, Lidow, 1995; Khan et al., 1998), very few studies have characterized the impact of the DAergic system on the visual cortex (Antal et al., 1997; Noudoost and Moore, 2011; Arsenault et al., 2013; Zaldivar et al., 2014). In monkeys, the systemic administration of D2 receptors antagonists during a visual discrimination task yielded alterations of components of V1 visual evoked potentials (Antal et al., 1997). Despite the evidence that the DAergic system influences the function of the primary visual cortex, the impact of dopamine receptors in the modulation of neuronal responses in the primary visual cortex to specific visual features (e.g., contrast response and SF tuning) remains unknown.

To shed more light on this issue, the present study investigated the contribution of D2 receptors in the organization and function of the primary visual cortex by studying cortical responses in an animal model lacking these receptors. In a first step, we confirmed the presence of D2 receptors in the primary visual cortex of mice. Then, we used optical imaging of intrinsic signals to assess the organization and function of primary visual cortex from D2-KO mice.

We found that the absence of D2 dopamine receptors influenced the cortical processing of spatial features in V1 of mice without alteration of the contrast response and cortical organization.

## Materials and methods

### Animals

Adult D2 receptor deficient mice (*n* = 12) (Kelly et al., 1997) and their control wild-type littermates (*n* = 9) were obtained from Jackson Laboratory (Bar Harbor, Maine). Mice were housed in a controlled environment with a 12 h light/dark cycle with food and water *ad libitum*. All procedures were carried out in agreement with the guidelines of the Canadian Council for the Protection of Animals, and the experimental protocol was approved by the Ethics Committee of the University of Montreal.

### Surgical procedures

Animals were anesthetized with an intraperitoneal injection of urethane (2 g/Kg, in saline). Atropine (0.05 mg/Kg) was injected subcutaneously to reduce tracheal secretion and to counteract the parasympathomimetic effects of the anesthesia. Injectable lidocaine (2%) was used at incision sites. Lidocaine gel was also used at all pressure points. In order to improve the animals' condition under prolonged anesthesia, a tracheotomy was performed (Moldestad et al., 2009). Animal core body temperature was maintained around 37°C using a heating pad feedback-controlled by a rectal thermoprobe. Viscous artificial tears were used when necessary to avoid corneal dehydration. Animals were placed in a stereotaxic apparatus and the scalp and connective tissues were removed to expose the occipital portion of the skull. A 10 mm wide metal ring was glued over the skull to serve as an imaging chamber. Low melting point agarose (1% in saline) was used to fill the chamber, which was then sealed with a glass cover slip. Electrocardiogram (ECG) and core body temperature were monitored throughout the experiment. After experiments, tissue samples were collected and individual genotypes were confirmed by PCR analysis.

### Visual stimuli

Visual stimuli were projected on a flat translucent screen at 21 cm from the animal's eyes covering 150 by 135 degrees of visual field. Stimuli were generated by the Vpixx software (version 2.8.9, Vpixx Technologies, Saint-Bruno, QC, Canada). Periodic stimulation consisted in full screen vertical or horizontal 2 degree thick white bars drifting over a black background in four directions (0, 90, 180, and 270 degrees) at 0.2 Hz for 10 min (**Figures 2C,E**) (Kalatsky and Stryker, 2003). In order to assess ocular dominance, the full-screen bar was replaced by a 10 × 2 degree bar that was presented along the elevation axis at the vertical meridian (Cang et al., 2005a). Episodic stimuli consisted in full-screen sinusoidal gratings drifting in four directions (0, 90, 180, and 270 degrees) at 2 Hz (**Figure 2G**). To evaluate contrast sensitivity, gratings at 0.02 cpd were shown at different contrasts (6, 12, 25, 50, and 100%). 100% contrast gratings with varying SFs (0.005 to 0.64 cpd) were used to assess the SF selectivity function and its cut-off values. Episodic trials lasted 20 s and consisted in the presentation of a uniform gray screen (blank stimulus) for 5 s, followed by 2 s of stimulation and a post-stimulus period of blank for 13 s. Trials were repeated 10 times, and conditions were randomly presented. Apart from ocular dominance tests, all stimuli were presented binocularly.

### Data acquisition and processing

Images were obtained with a cooled 12-bit CCD camera (Dalsa 1M60, Colorado Springs, USA) coupled to a macro lens (Nikon, AF Micro Nikkor, 60 m, 1:2.8 D). Images were sampled at 2 Hz for experiments involving episodic stimulation and 1 Hz for retinotopic maps with a resolution of 512 × 512 pixels. Data acquisition was controlled by an Imager 3001 system and with VDAQ software (Optical Imaging Ltd., Rehovot, Israel). An anatomic reference image was taken under a 550 nm illumination for optimal contrast between the cortical matter and the blood vessels (**Figures 2A,B**). The focus was then set to approximately 300 μm deep from the cortical surface and intrinsic signals were acquired under 630 nm illumination. The imaged area encompassed the primary visual cortex of both hemispheres. The analysis was performed using custom scripts in MATLAB (The Mathworks, Natick, MA).

Cortical retinotopic maps were obtained from the spectral decomposition of data originating from periodic stimulation trials (Kalatsky and Stryker, 2003). This resulted in frequency power spectrum maps, accompanied by their respective phase maps. Retinotopic maps were created by the product of the phase component and amplitude of the periodic intrinsic signal (**Figures 2C–F**). Regions of interest (ROIs) delimiting each primary visual cortex were manually drawn based on the cortical activation maps as in Groleau et al. (2014) and Farishta et al. (2015). Different parameters drawn from both the amplitude and phase components of the retinotopic maps were used to trace a profile of cortical organization. The ROIs served to assess the cortical surface and shape, while the phase component comprised in the respective ROIs was used to assess the cortical magnification factor (CMF), scatter, and the extent of the visual field represented.

The shape of primary visual cortex was assessed by fitting ellipses to ROIs using the MATLAB built-in function *regionprops*. The ellipsis eccentricity was used as an “ovality index” in order to establish a parameter of cortical shape. This parameter provides an elongation index (ranging from 0 to 1) of the fitted ellipsis in which higher values represents more elongated ellipses. The phase scatter was calculated on a pixel-to-pixel basis by the subtraction of the phase value of each pixel by the mean phase of its 25 neighboring pixels. The scatter index represents the standard deviation of the mean phase scatter for the ROI and it is a measure of the “quality” of the retinotopy in which lower scatter values represent more uniform phases with “smoother” transitions (Cang et al., 2005a). The extent of cortical representation of the visual field, named here the apparent visual field, was calculated by fitting a Gaussian curve to the phase span for azimuth and elevation maps. The apparent visual field is represented by the 95% confidence interval of the curve and is expressed in degrees. The CMF was also drawn using the same above-mentioned procedures. CMF was determined as the distance between the centroid of the majoritarian phase and the centroid of the sigma from the Gaussian and it is expressed in millimeters per degree.

The ocular dominance index (ODI) was determined as described by Cang et al. (2005a). In brief, ipsilateral and contralateral retinotopic maps were generated from the stimulation of the central visual field. Ocular dominance values were calculated for each pixel using the following operation: (C – I) / (C + I), where C and I represent the amplitude values for the contralateral and ipsilateral stimulation paradigms respectively. The ODI was obtained by averaging the ocular dominance values from each pixel.

Responses from the episodic stimulation paradigm were used to assess the contrast response function and SF selectivity curve (**Figures 2G,H**). As done for the retinotopy, ROIs were manually traced on the amplitude maps obtained at optimal conditions (i.e., 100% contrast or 0.02 cpd, for contrast and SF respectively). The responses to the four drifting directions were averaged. Signal amplitude was calculated on a pixel-to-pixel basis and subsequently averaged across the ROI. A modified Naka-Rushton function (Equation 1) was used to fit the contrast responses, in which *n* is the exponent (slope coefficient), and C_50_ is the contrast corresponding to the half of the maximum response amplitude.

(1) $$\begin{array}{l} z=\frac{C^{n}}{C_{50}^{n}+\;C^{n}} \end{array}$$

The spatial selectivity curve was obtained by the fitting of data with an asymmetric Gaussian curve (Equation 2), in which *e* is the Euler's constant, s is the standard deviation, p is the optimal SF and *o* is the log offset.

(2) $$\begin{array}{l} z=e^{\frac{-1}{\;2s^{2}}{\;}^{*}\log{\;\left(\frac{x+o}{p+o}\right)}^{2}} \end{array}$$

The SF high cut-off was considered as the SF value at which the model intercepts the level of noise present in the blank recordings from each neuron.

### Statistics

Kolmogorov-Smirnov test was used to characterize data distributions. When data were normally distributed, Student's *t*-tests were performed, otherwise Wilcoxon Rank-sum tests were used. All results are presented as mean ± SEM, unless otherwise stated.

In order to compare the contrast and SF sensitivity response curves fitted to the datasets from D2-KO mice and their WT littermates, *F*-tests were performed. In brief, the *F*-test compares the sum of squared errors of prediction (SSE) from the curve fit of the control and D2-KO groups with values obtained from a curve fit to the pooled data. If the two datasets come from different populations, the SSE from the pooled data fit should increase and the null hypothesis is rejected. In case of a significant difference between the curve fits, Welch's *t*-test was performed on the model parameters (as shown in Equations 1 and 2). The levels of significance are indicated as follows: ^*^p < 0.05, ^**^p < 0.01 and ^***^p < 0.001.

### Expression of D2 receptors in visual cortex

**Figure 1:**
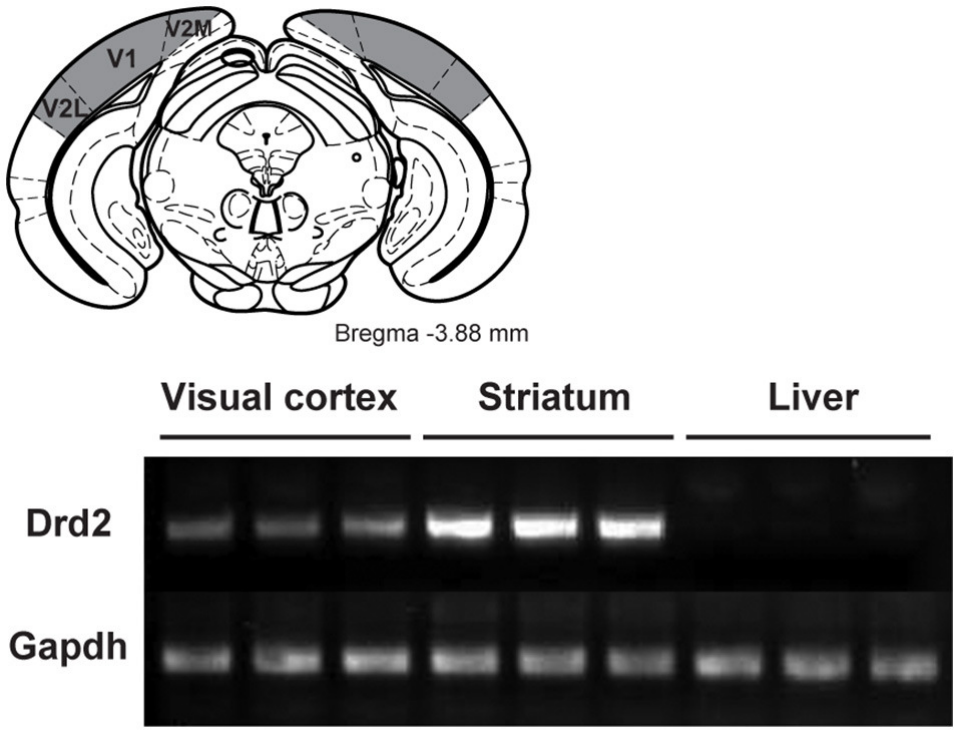
Characterization of the expression of D2 receptors in the visual cortex of wild-type mice. **(Top)** Location of the visual cortex (gray areas) extracted for the quantitative real time PCR (qRT-PCR) analysis (Adapted from Paxinos and Franklin, 2008). **(Bottom)** qRT-PCR for Drd2 and GAPDH from visual cortex, striatum and liver tissues. GAPDH was used as a loading control. V1, primary visual cortex; V2L, secondary visual cortex lateral area; V2M, secondary visual cortex medial area.

A subgroup of WT mice was used to investigate the presence of D2 receptors in V1. Mice were killed by a rapid cervical dislocation. Heads of animals were immediately cooled by immersion in liquid nitrogen for 6 s. The brains were extracted and 500 μm thick serial coronal sections were prepared using ice-cold adult mouse brain slicer and matrix (Zivic instruments). Visual cortices (Figure 1), striatum and liver tissues were dissected rapidly (within 90 s) on an ice-cold surface using microsurgical knife (KF Technology) and frozen in liquid nitrogen (one mouse per sample). Tissue samples were lysed by adding TRI reagent (Zymo research) and RNA was extracted by Direct-zol RNA kit according to the manufacturer's instructions (Zymo research). RNA concentration was quantified using ND-1000 Spectrophotometer (NanoDrop Technologies).

Complementary DNA was synthesized using a reverse transcriptase SuperScript III kit according to the manufacturer's instructions (Invitrogen). PCR was performed using following primers: Drd2 forward—TACGTGCCCTTCATCGTCAC, Drd2 reverse—CCATTGGGCATGGTCTGGAT, Gapdh forward—ACAGTCCATGCCATCACTGCC, Gapdh reverse—GCCTGCTTCACCACCTTCTTG.

## Results

### Receptor expression

Quantitative real time PCR was used to determine the presence of D2 receptors. Figure 1 shows the presence of the receptor in the visual cortex comprising V1 and adjacent visual areas. As expected a strong band was observed in the striatum while no receptors were seen in the liver.

### Anatomo-functional maps

**Figure 2:**
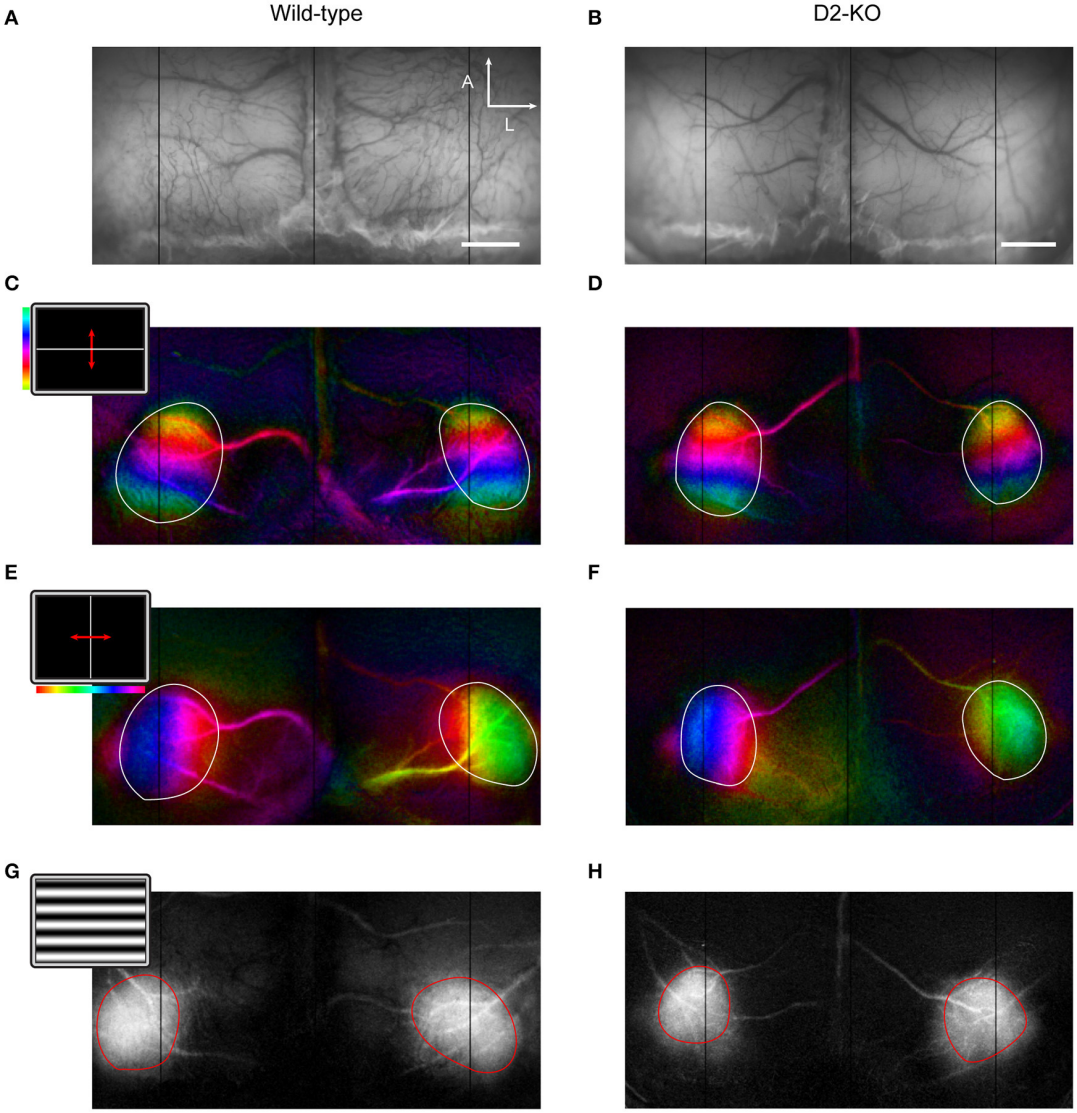
Retinotopic maps in V1 of wild-type and D2-KO mice. **(A,B)** Anatomical reference images of cortical vessels at 545 nm illumination (scale bar = 1 mm; A, anterior; L, lateral). **(C–F)** Retinotopic maps along elevation and azimuth. The insets in **(C,E)** show the stimulus which consisted of a two degrees white bar drifting periodically at 0.2 Hz over a black background. The color bar indicates the position of the bar along the horizontal or vertical axis. The visual field covered in elevation and azimuth was 135 and 150 degree, respectively. **(G,H)** Cortical activation maps to episodic stimulation (inset in **G**, drifting gratings at 100% contrast). Regions of interest (ROIs) are represented by the white and red lines.

In the present study, optical imaging of intrinsic signals was used to assess the cortical architecture from D2-KO mice. Retinotopic cortical maps were obtained using the periodic stimulus paradigm described by Kalatsky and Stryker (2003). Representative examples of azimuth and elevation cortical maps from D2-KO and WT mice are shown in Figures 2C–F. Initially, the qualitative analysis of the maps did not reveal any obvious alterations in cortical morphology (V1 shape) or visuotopic representation (number of phases) of D2-KO mice. Further analysis was performed on the amplitude and phase components of the cortical retinotopic maps in order to respectively quantify different features of the cortical morphology and functional organization. Our results are summarized in Table 1. V1 boundaries were drawn from the amplitude component of the retinotopic maps from which the cortical surface and ovality index were analyzed. The surface of V1 in D2-KO mice was not significantly different to that of their WT littermates. Similarly, no differences were observed between the ovality index of both groups. The functional organization of V1 from D2-KO mice was also examined. First, the extent of the visual field stimulated (apparent visual field) was assessed. D2-KO mice exhibited apparent visual field values similar to those of WT mice in both azimuth and elevation. The phase component was equally used to quantify the CMF (see Materials and Methods) for azimuth and elevation maps. No significant differences between CMF values of D2-KO and WT mice were observed, indicating that the lack of D2 receptors had no effect on the amount of cortical surface dedicated to the processing of a specific part of the visual field. Finally, the scatter index from D2-KO mice was computed. Again, no significant differences were noted between the quality of the retinotopic maps derived from azimuth and elevation phase maps of D2-KO and WT mice. Taken together, the analysis of cortical retinotopic maps from D2-KO mice revealed that the lack of D2 dopamine receptors had no significant impact on the functional organization of V1.

**Table 1:** Summary of analysis performed on wild-type and D2-KO mice azimuth and elevation retinotopic maps.

**Parameters analyzed**	**D2-KO**	**Wild-type**	***p*-value**
**AZIMUTH**			
Activated cortical surface (mm^2^)	3.956 ± 0.0269	3.816 ± 0.0385	*p* = 0.357
Ovality index	0.5469 ± 0.0046	0.5673 ± 0.0071	*p* = 0.298
Apparent visual field (^◦^)	37.81 ± 0.7079	40.46 ± 1.2141	*p* = 0.417
Cortical Magnification Factor (mm/^◦^)	0.03404 ± 0.001	0.03243 ± 0.0023	*p* = 0.7908
Scatter index (^◦^)	43.94 ± 1.2142	53.62 ± 2.2030	*p* = 0.179
**ELEVATION**			
Activated cortical surface (mm^2^)	3.971 ± 0.0271	3.8 ± 0.0523	*p* = 0.099
Ovality index	0.5778 ± 0.0050	0.5645 ± 0.0072	*p* = 0.747
Apparent visual field (^◦^)	81.96 ± 0.8394	77.89 ± 1.0863	*p* = 0.3328
Cortical Magnification Factor (mm/^◦^)	0.0298 ± 0.0003	0.03064 ± 0.0006	*p* = 0.5856
Scatter index (◦)	39.46 ± 0.6573	47.38 ± 1.6127	*p* = 0.398

**Figure 3:**
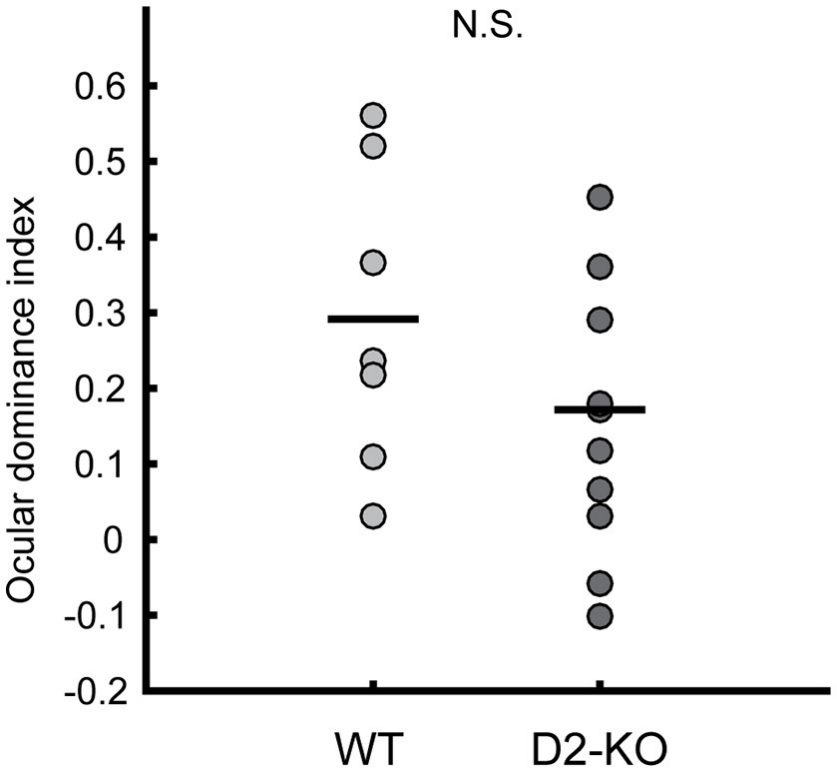
Scatter plot of D2-KO and WT ocular dominance indices (ODIs). No significant differences were observed between groups (Wilcoxon rank sum test, *p* = 0.2109). Black bars represent the mean. N.S.: not significant.

Further, the ocular dominance of D2-KO and WT mice was assessed by calculating the ocular dominance index (ODI). Figure 3 shows the distribution of ODI values for D2-KO mice and their WT littermates. ODI values of D2-KO mice were not different from WT mice (0.173 ± 0.056 vs. 0.29 ± 0.07, Wilcoxon rank sum test, *p* = 0.21), indicating that the ocular dominance was not affected by the congenital lack of D2 dopamine receptors.

### Response properties

In a next step, we measured and compared the SF selectivity and the contrast response of V1 from WT and D2-KO mice using drifting sinusoidal gratings.

#### Contrast response function

**Figure 4:**
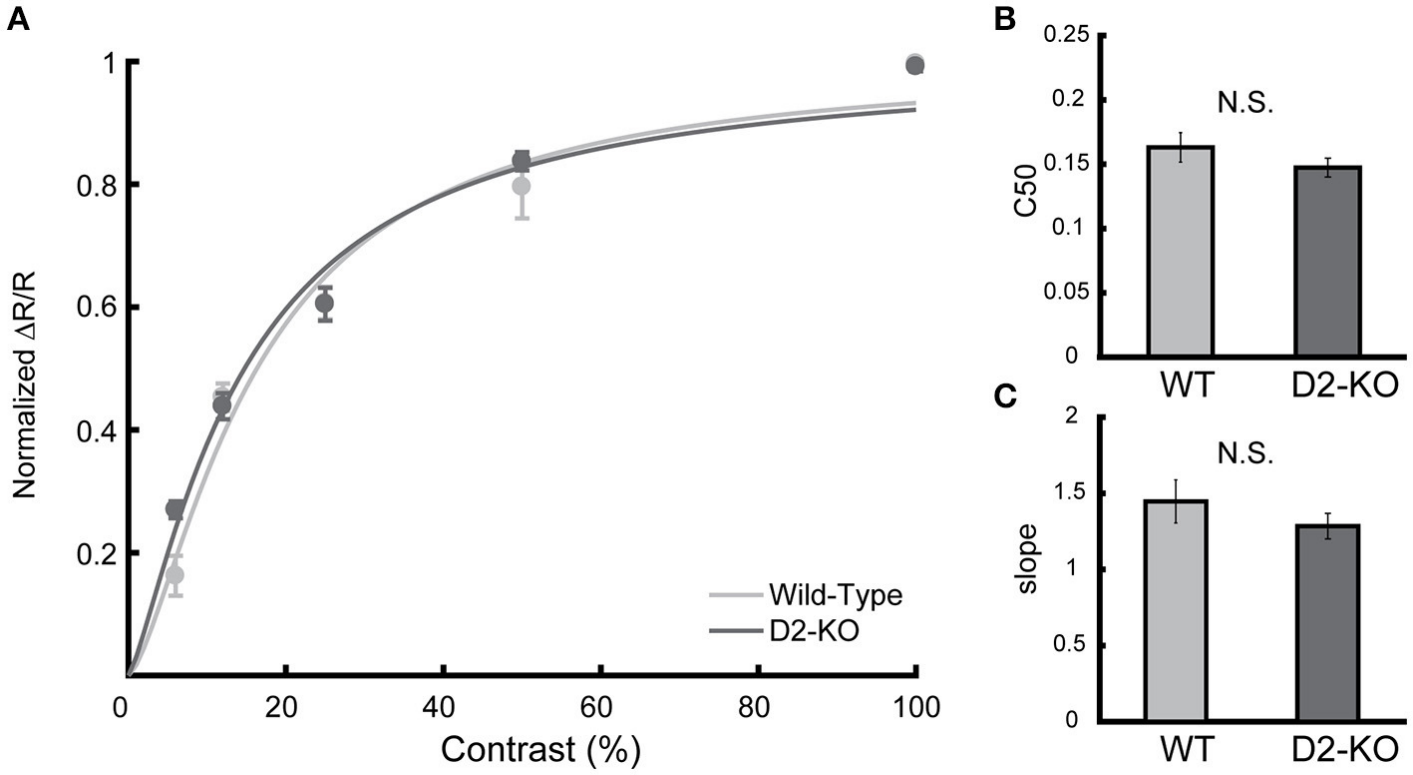
Contrast response curve for D2-KO and wild-type mice V1. **(A)** Curve fitted on normalized response amplitude (mean ± SEM) of pooled individual data elicited by gratings of varying contrasts (6, 12, 50, and 100%). **(B,C)** Comparison of parameters drawn from the curve fits, c50 and slope coefficient respectively. No significant difference was observed between the datasets (*F*-test, *p* = 0.27). N.S.: not significant.

Contrast response curves were obtained from V1 of D2-KO and WT mice. Individual datasets were pooled, and data were fitted in order to obtain a response curve for each group. Figure 4 shows the contrast response curves for D2-KO and WT mice as well as the comparison of the C50 values and slope coefficient drawn from the curve fits. While V1 of D2-KO mice tended to be less sensitive to contrast (lower C50 and slope) than WT mice, this trend, however, did not reach statistical significance. Indeed, F-statistics performed on the curve fits failed to reveal any differences between the two curves (*F*-test, *p* = 0.27) and comparison of C50 and slope coefficient values (Welch's *t*-test) revealed no significant differences between the response to contrast of D2-KO and WT mice.

#### Spatial frequency selectivity

**Figure 5:**
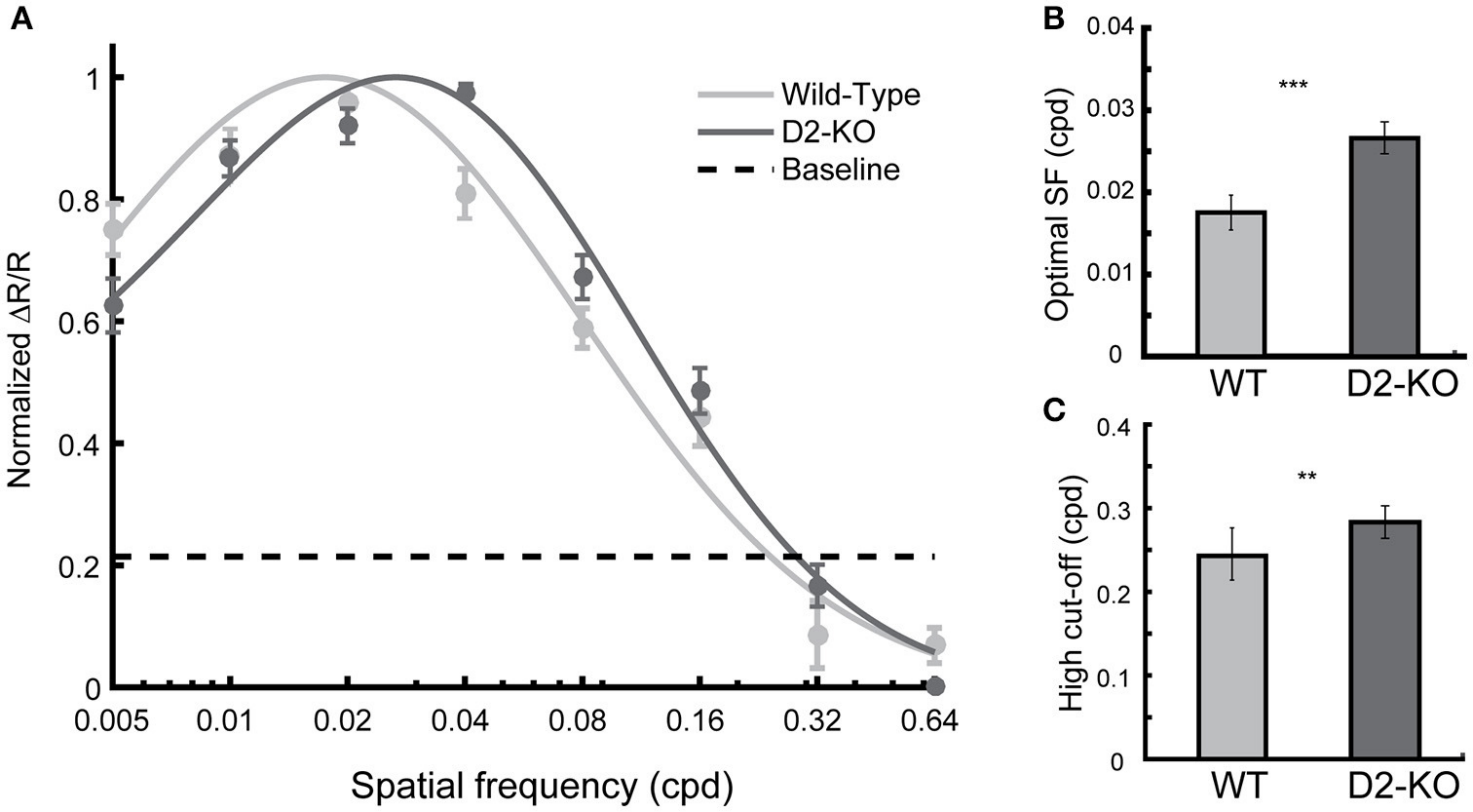
Spatial frequency (SF) tuning function for D2-KO and wild-type mice V1. **(A)** The normalized response amplitude (mean ± SEM) of pooled individual data is plotted against the SFs tested. Note that the D2-KO mice curve is shifted toward higher SFs. The curves are statistically different (*F*-test, *p* < 0.05). **(B)** SF eliciting the maximal response (mean ± SEM) from V1 of D2-KO and wild-type. V1 neurons of D2-KO were optimally activated by higher SFs compared to wild-type mice (Welch's *t*-test, ^***^p < 0.001). **(C)** Predicted high cut-off for V1 of D2-KO and wild-type mice. The SF threshold was determined by the value at which the model intercepts the level of noise (example of cut-off level depicted in **A**) present in the blank recordings for each individual (Students *t*-test, ^**^p < 0.01).

The overall SF selectivity of V1 was also evaluated. As for contrast response functions, datasets in each group were pooled and curve fits were applied to obtain SF selectivity curves (Figure 5A). One may note that SF tuning function of D2-KO mice was shifted toward higher SFs when compared to the WT curve (*F*-test, *p* < 0.05). This was accompanied by changes in the optimal SF and high cut-off. D2-KO mice exhibited higher optimal SF (Figure 5B; 0.026 ± 0.009 vs. 0.0175 ±0.0021, Welch's *t*-test, *p* < 0.001) and high cut-off (Figure 5C; 0.28 ± 0.019 vs. 0.24 ± 0.031, Welch's *t*-test, *p* < 0.01) compared to their WT littermates. Thus, the SF selectivity profile of D2-KO mice revealed that the lack of functional D2 dopamine receptors increased the sensitivity of V1 neuronal populations by shifting the optimal SF and increasing the SF high-cut-off.

## Discussion

In this study, taking advantage of knockout animal models, we present the first evidence of altered visual responses in the primary visual cortex of mice lacking functional D2 dopamine receptor. The main impact of the lack of these receptors was a significant change in the SF tuning function. This change was not accompanied by a modification of the response to the stimulus contrast. Further, the analysis of retinotopic maps indicated that V1 shape or retinotopic organization, as well as ocular dominance in binocular cortex were not altered in D2-KO mice.

### Functional structure of D2-KO mice V1

In order to investigate if the lack of D2 receptor function could cause structural alterations in mice primary visual cortex, the present study analyzed the retinotopic maps obtained by the periodic paradigm described by Kalatsky and Stryker (2003). Previous studies from us and others have successfully applied this technique in order to assess the impact of different molecular pathways and receptors on the structural organization of V1 connectivity in different knockout mice models (Cang et al., 2005b; Groleau et al., 2014; Farishta et al., 2015). In the present study, the analysis of visual maps revealed that the cortical structure of D2-KO mice did not differ from their WT littermates, suggesting that D2 receptors are not involved in V1 structural organization. Furthermore, the lack of functional D2 dopamine receptors did not affect the ocular dominance in V1 of D2-KO mice.

DAergic receptors are known to play a role in brain development (see Money and Stanwood, 2013 for a review) and pharmacological approaches as well as knockout models have been extensively used to investigate the impact of the lack of these receptors in cortical structure (Jones et al., 2000; Stanwood et al., 2005; Zhang et al., 2010). In a D1-KO mouse model, Stanwood et al. (2005) observed that the lack of functional D1 receptors induced morphological alterations in the dendritic projection of the prefrontal and anterior cingulate cortex whereas the cellular morphology of neurons from the visual cortex remained intact, suggesting that the role of D1 receptors in development is confined to cortical areas with major DAergic input, such as the prefrontal cortex, without any detectable alterations in cortical circuitry of visual areas.

Our data shows that the absence of D2 receptors did not alter the cortical organization of V1 (as shown by the retinotopy and ocular dominance), suggesting that, as observed for D1 receptors, D2 receptors do not play a preponderant role in the organization of the circuitry in the visual cortex. However, given the experimental approach used here, we cannot rule out any morphological changes occurring at the cellular level or in other parts of the visual pathways. Although no structural alterations were observed in V1 of D2-KO mice, the lack of function of D2 receptors did influence cortical processing as described in the next section.

### Contrast and SF functions of D2-KO mice V1

Visual areas are generally considered to have a less prominent DAergic innervation compared to other parts of the brain such as the prefrontal cortex (Papadopoulos and Parnavelas, 1990; Boumghar et al., 1997; Zhao et al., 2001; Govindaiah and Cox, 2005, 2006). Nevertheless, there is evidence that DA and its receptors are implicated in different aspects of the processing of visual information from the retina to the cortex (Papadopoulos and Parnavelas, 1990; Antal et al., 1997; Witkovsky, 2004; Lavoie et al., 2013; Zaldivar et al., 2014). In particular, previous studies have demonstrated modulatory effects of DAergic receptors on SF processing and contrast response function in the retina and visual thalamus, respectively (Zhao et al., 2001; Witkovsky, 2004). One could thus hypothesize that V1 neurons will exhibit a similar modulation of the contrast response by inheriting the effects produced in the LGN relay neurons. Our findings do not support this assumption since the contrast response function of D2-KO mice was not significantly different from their WT littermates, indicating that D2 receptors do not influence the contrast sensitivity of V1 neurons. This result is thus at odds with Zhao et al. (2001) report that the injection of D2 receptor agonists provoked a facilitation or inhibition of the contrast response gain of relay neurons in the cat dLGN in a dose-dependent manner. It is worth emphasizing that the contrast responses obtained in the present study is the mean response of the whole primary visual cortex. Since D2 receptors activation gives rise to both facilitation and suppression in the thalamus (Zhao et al., 2001), it is possible that those modulatory effects are balanced or even nullified once the different thalamic signals are integrated in the cortex, avoiding any profound effects on the cortical processing of contrast. Alternatively, a recent study in mice indicated that contrast adaptation in V1 arises primarily from the local circuitry, with less contribution from the thalamus (King et al., 2016). Hence, the compensatory mechanisms from local circuitry of V1 may dampen the potential modulatory D2-mediated changes observed in the thalamus on the contrast response.

There is little evidence of the specific role of D2 dopamine receptors on the modulation of the SF selectivity of neurons in the visual system. Until now, the effects of the DAergic system on the SF responses have been observed mostly at the retinal level. For instance, in anesthetized monkeys, the administration of l-sulpiride, a selective D2 receptor antagonist caused a reduction of the amplitude of the pattern-ERG (PERG) at the optimal SF (Tagliati et al., 1994). Similar effects were also reported in humans (Stanzione et al., 1995). In our study, the lack of D2 dopamine receptors induced an increased sensitivity to SF in V1, with D2-KO mice exhibiting higher optimal SFs and cut-off values. To our knowledge, this is the first demonstration of the impact of the lack of D2 dopamine receptors on the SF selectivity in V1. Aside obvious methodological differences between our study and the above-mentioned ones, our data suggest that the effects of the lack of D2 dopamine receptors on V1 SF tuning do not arise from the retina. Therefore, it is most likely that the increased SF sensitivity presented in D2-KO mice resulted from changes occurring in the visual thalamus or directly in the cortex since D2 receptors are present in the LGN (Zhao et al., 2001) and in the visual cortex, as revealed here. Nonetheless, it is worth noting that the animal model used in the present study lacks congenitally the D2 dopamine receptor and this takes place ubiquitously. Therefore, the effects observed in the primary visual cortex may result from changes arising at the subcortical level and/or in higher-order visual areas.

## Conclusion

Our data shows that the lack of function of D2 receptors does not impair the structural organization of neuronal populations of V1. However, compared to WT littermates, D2-KO mice were characterized by an increase of the response amplitudes to higher SFs and of the high SF cut-off. These results suggest that D2 receptors are specifically implicated in the processing of spatial aspects of the visual scene in V1.

## Author contributions

BS: performed the surgical procedures, part of data acquisition, performed data analysis and wrote the manuscript; MA: performed part of data acquisition; CQ: performed the PCR experiments; JB: provided the knockout mice and participated in the formulation of original scientific questions, study design and manuscript corrections; CC: participated on the formulation of original scientific questions, on the study design, provided guidance throughout the experiment, and provided helpful feedback on the manuscript writing.

### Conflict of interest statement

The authors declare that the research was conducted in the absence of any commercial or financial relationships that could be construed as a potential conflict of interest.
